# High-Strength Nanocomposite Hydrogels with Swelling-Resistant and Anti-Dehydration Properties

**DOI:** 10.3390/polym10091025

**Published:** 2018-09-14

**Authors:** Bo Xu, Yuwei Liu, Lanlan Wang, Xiaodong Ge, Min Fu, Ping Wang, Qiang Wang

**Affiliations:** 1 Key Laboratory of Eco-Textile, Ministry of Education, College of Textile and Clothing, Jiangnan University, Wuxi 214122, China; boxu@jiangnan.edu.cn (B.X.); 18861822651@163.com (Y.L.); 15852776701@163.com (L.W.); 18861870698@163.com (X.G.); wxwping@163.com (P.W.); 2 College of Chemical and Environmental Engineering, Shandong University of Science and Technology, Qingdao 266590, China; fuminis@163.com

**Keywords:** nanocomposite hydrogels, alumina, high-strength, anti-dehydration, swelling resistance

## Abstract

Hydrogels with excellent mechanical properties have potential for use in various fields. However, the swelling of hydrogels under water and the dehydration of hydrogels in air severely limits the practical applications of high-strength hydrogels due to the influence of air and water on the mechanical performance of hydrogels. In this study, we report on a kind of tough and strong nanocomposite hydrogels (NC-G gels) with both swelling-resistant and anti-dehydration properties via *in situ* free radical copolymerization of acrylic acid (AA) and *N*-vinyl-2-pyrrolidone (VP) in the water-glycerol bi-solvent solutions containing small amounts of alumina nanoparticles (Al_2_O_3_ NPs) as the inorganic cross-linking agents. The topotactic chelation reactions between Al_2_O_3_ NPs and polymer matrix are thought to contribute to the cross-linking structure, outstanding mechanical performance, and swelling-resistant property of NC-G gels, whereas the strong hydrogen bonds between water and glycerol endow them with anti-dehydration capacity. As a result, the NC-G gels could maintain mechanical properties comparable to other as-prepared high-strength hydrogels when utilized both under water and in air environments. Thus, this novel type of hydrogel would considerably enlarge the application range of hydrogel materials.

## 1. Introduction

Hydrogels are polymeric materials that consist of three-dimensional (3D) networks and a large amount of water [1]. Because of their well-known hygroscopicity, hydrophilicity, biocompatibility, and similarity to native tissues, hydrogels have been widely used in a wide range of applications, including as super-absorbents, tissue engineering, drug delivery, wound dressing, as well as cell culture [2,3,4,5,6]. Recently, hydrogels with excellent mechanical properties have attracted increasing attention due to their potential for use in artificial tissues, bio-actuators, soft robots, and so forth [7,8,9,10,11]. Thus, massive efforts have been made to improve the mechanical properties of hydrogels [12]. Despite several tough and strong hydrogels being fabricated via different strategies [13,14,15,16,17,18], the practical applications of these hydrogels are still experiencing challenges. On the one hand, as a kind of “soft and wet” material, hydrogels are usually utilized in under water environments. Unfortunately, most hydrogels would absorb water to swell as a result of the difference in osmotic pressure [19]. After swelling, the strength and toughness of hydrogels are drastically weakened. On the other hand, when used in air environments, water is inevitably lost from the hydrogels by evaporation, leading to the drying-out of hydrogels, which also causes degradation of the softness of hydrogels. Therefore, developing hydrogel materials with excellent mechanical properties as well as swelling-resistance and anti-hydration properties is an attractive but highly challenging task. Recently, several research groups have made some outstanding contributions to this area. For example, Sakai et al. reported a high-strength and non-swelling hydrogel with a homogeneous network structure by random copolymerization of hydrophilic and thermoresponsive monomers with tetra-armed polyethylene glycol (PEG) backbone [19]. When the thermoresponsive segment ratio is 0.4, the resultant hydrogels swelled at 10 °C but could recover its original shape at around 37 °C. In another study, Jin et al. fabricated a type of dual-cross-linked (DC) hydrogels composed of tannin acid (TA) cross-linked poly(vinyl alcohol) and chemically cross-linked polyacrylamide. A strong multiple polymer—TA hydrogen bonds not only endow hydrogels with tough mechanical properties, but also suppress the swelling of hydrogels, resulting in good swelling resistance [20]. Our previous studies also demonstrated that hydrogels with low swelling ratios could be realized using titania (TiO_2_) and alumina (Al_2_O_3_) nanoparticles as inorganic cross-linking agents [21,22]. The low swelling ratios of these gels were supposed to contribute to the strong complexation interaction between nanoparticles and carboxyl groups on the polymer chains. Although these above-mentioned hydrogels achieved non-swelling or low swelling via different mechanisms, few reports have concentrated on hydrogels with anti-dehydration properties, not to mention that hydrogels possess both of these unique properties.

In this study, we successfully developed a novel high-strength nanocomposite hydrogels (NC-G gels) that simultaneously possess anti-dehydration and swelling-resistant properties, via *in situ* free-radical copolymerization of acrylic acid (AA) and *N*-vinyl-2-pyrrolidone (VP) in a water-glycerol bi-solvent containing small amounts of alumina nanoparticles (Al_2_O_3_ NPs) as the inorganic cross-linking agents. The obtained hydrogels exhibited excellent mechanical properties comparable to previously reported nanocomposite hydrogels. More importantly, the introduction of glycerol endows the obtained hydrogels with anti-dehydration ability in air due to the strong hydrogen bonding interactions between water and glycerol, which help to lock the water inside the polymer networks. Besides, slight shrinkage instead of swelling was observed when the NC-G gels were immersed in the water environment, which probably contributed to the increased cross-linking density of hydrogels. Due to unique anti-dehydration and swelling-resistant properties, the NC-G gels maintained outstanding softness and high mechanical properties both under water and in air environments, which could greatly expand the practical applications of hydrogels.

## 2. Materials and Methods

### 2.1. Materials

Acrylic acid (AA, >99%) and *N*-vinyl-2-pyrrolidone (VP, 99%) were purchased from Aladdin Reagent Co., Ltd., Shanghai, China. Glycerol (99.5%) was obtained from Innochem Co., Ltd., Beijing, China. 2-hydroxy-4′-(2-hydroxyethoxy)-2-methylpropiophenone (Irgacure 2959, 98%) was purchased from Energy Chemical Co., Ltd., Shanghai, China. A colloid solution of Al_2_O_3_ NPs with particle size of 10–20 nm and solid content around 10% was provided by Wanjing New Materials Co., Ltd., Hangzhou, China. Organic cross-linker *N*,*N*′-methylenebis-(acrylamide) (MBA, 98%) was obtained from Alfa Aesar Co., Ltd., Shanghai, China. All the reagents were used as received without further treatment.

### 2.2. Preparation of Hydrogels

The hydrogels were prepared by photo-initiated *in situ* free radical polymerization of AA and VP in the mixed solutions containing water, glycerol, and small amounts of Al_2_O_3_ NPs, following the procedure similar to our previous report [22]. Firstly, homogeneous precursor solutions were obtained by mixing a certain amount of raw Al_2_O_3_ colloid solution, deionized water, glycerol, and 20 mg Irgacure 2959 in a 20 mL glass bottle under a nitrogen atmosphere. Then, the precursor solutions were transferred into different molds after being degassed by a vacuum. Finally, free radical polymerization was conducted under 365 nm ultraviolet (UV) light (Scientz03-II, Xinzhi Co., Ltd., Ningbo, China) with the intensity of 60 mW/cm^2^ for 30 min. The resultant hydrogels were termed as NC-G*x* gels, in which *x*% represents the mass percentage of glycerol in the total water-glycerol solution. The molar ratio of AA to VP was fixed at 3:7 based on our experiment, since the hydrogel prepared with the monomer ratio of 3:7 exhibited the best comprehensive mechanical properties compared to its counterparts obtained with other monomer ratios. Unless otherwise specified, the total monomer content and the Al_2_O_3_ concentration in the water-glycerol solutions were fixed at 3 mol/L and 2%, respectively, although they can be altered within a large range in order to meet the requirements of different applications. For comparison, the conventional hydrogel cross-linked by 30 mg organic cross-linker MBA was also prepared and named OR-G gels. The detailed sample names and the corresponding compositions are listed in Table 1.

### 2.3. Evaluation of Anti-Dehydration and Swelling-Resistant Properties

The anti-dehydration and swelling-resistant properties of the hydrogels were evaluated in air atmosphere (the temperature and humidity were fixed at 27 °C and 60%, respectively) and pure water (water was changed every 12 h). The sheet-like samples with size of 10 × 10 × 2 mm were placed in the above-mentioned circumstances and weighed after 10 days. The weight variations were used to evaluate their anti-dehydration and swelling-resistant properties. The weight variation was calculated as follows:

Weight variation (%) = (*W*_0_ – *W*_10_)/*W*_0_ × 100% (1)

where *W*_0_ and *W*_10_ are the weights of the samples before measurement and after 10 days, respectively. Three measurements were conducted for each hydrogel, and the average values were used for discussion.

### 2.4. Mechanical Tests of Hydrogels

The mechanical properties of hydrogels were tested on a MIT-1 universal test machine (Sanfeng Co., Changzhou, China) with a 50 N load cell. The as-prepared sheet-like samples with size of 10 × 50 × 2 mm were clamped between two clamps with a gauge length of 10 mm. Then the tensile tests were conducted at a speed of 100 mm/min until fracture under ambient conditions (about 25 °C). The fracture stress was calculated by the force at the fracture point divided by the initial cross-sectional area of the hydrogel sample (ca. 20 mm^2^). The elastic modulus was calculated from the slope over 50–100% of strain on the stress-strain curve. The fracture stress, elongation at break, and elastic modulus of hydrogels were used for discussion. As for the samples placed in air and under water environments, the as-prepared samples with the size mentioned above were placed under certain conditions described in Section 2.3. followed by the mechanical tests, and their values for mechanical properties were calculated based on their actual sizes after 10 days.

### 2.5. Characterization of Hydrogels

Fourier transform infrared (FTIR) spectra of Al_2_O_3_ NPs, neat copolymer of AA and VP, as well as NC-G0 gel were obtained using an IRAffinity-1S (Shimadzu Co., Kyoto, Japan) FTIR spectrometer with an attenuated total reflectance (ATR) accessory. All of the samples were dried in a 95 °C oven before conducting the FTIR characterization. Scanning electron spectroscopy (SEM) was conducted to observe the microstructures of the NC-G gels. The as-prepared hydrogels were first placed in water (water was changed every 12 h) for 10 days to replace the glycerol with water. Then, the samples were immersed in liquid nitrogen and followed by freeze-dry (FD-1A-50, Boyikang Co., Beijing, China) for 48 h at −48 °C. The fractured surfaces were observed using a Quanta 250 (FEI Co., Hillsboro, OR, USA) instrument at an acceleration voltage of 20 kV after coating with gold. The thermal gravimetric analysis (TGA) and differential scanning calorimetry (DSC) measurement of NC-G gels were performed on a Pyris 1 (Perkin Elmer Co., Waltham, MA, USA) and DSC 8000 (Perkin Elmer Co., Waltham, MA, USA) instruments, respectively. For both of the characterizations, the heating rate was set at 10 °C/min from 30 to 300 °C under N_2_ atmosphere.

## 3. Results and Discussion

### 3.1. Formation of Nanocomposite Hydrogels (NC-G Gels)

**Scheme 1:**
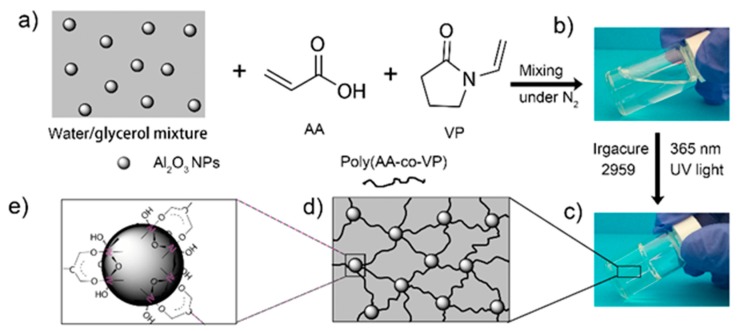
Preparation and cross-linking mechanism of NC-G gels. (**a**) Materials for the preparation of NC-G gels. Digital pictures of (**b**) hydrogel precursor and (**c**) NC-G gels. (**d**) Illustration of the network structure NC-G gels. (**e**) Proposed cross-linking mechanism of NC-G gels.

**Figure 1:**
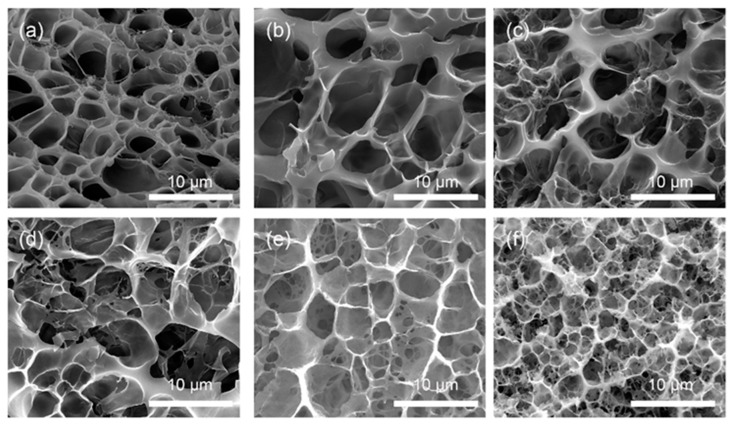
The typical scanning electron microscopy (SEM) images of NC-G and OR-G gels. (**a**) NC-G0, (**b**) NC-G20, (**c**) NC-G40, (**d**) NC-G60, (**e**) NC-G80, and (**f**) OR-G60.

Hydrogels have been extensively applied in various areas due to their specific physicochemical characteristics [23,24,25]. However, the poor mechanical properties of hydrogels has severely limited their application range. Although some tough and strong hydrogels have been prepared via different strategies, their outstanding mechanical performance can only be exhibited in their as-prepared states. Notably, hydrogels are generally utilized either in air or water environments. Under these circumstances, hydrogels would lose or absorb a large proportion of water within their polymer networks, which inevitably influence their mechanical properties, limiting their practical applications. To address this problem, in this study, we developed a novel alumina cross-linked bi-solvent NC-G gel, which possesses both anti-dehydration and swelling resistant properties. The NC-G gels were prepared by a feasible photo-initiated free radical copolymerization of AA and VP in mixtures of water and glycerol containing small amounts of Al_2_O_3_ NPs as illustrated in Scheme 1. It was found that all of the obtained NC-G gels, regardless of their glycerol content, were uniform and stable, and could not be dissolved in water, indicating the formation of effective cross-linking structures. To confirm this hypothesis, SEM was conducted and the results are shown in Figure 1. It can be seen that all the NC-G gels and OR-G60 gel possess well-defined porous microstructures with the pore size around several micrometers, regardless of the glycerol content. These results confirmed that the introduction of glycerol in the precursors would not influence the monomer polymerization as well as the formation of cross-linked 3D polymer network structures.

**Figure 2:**
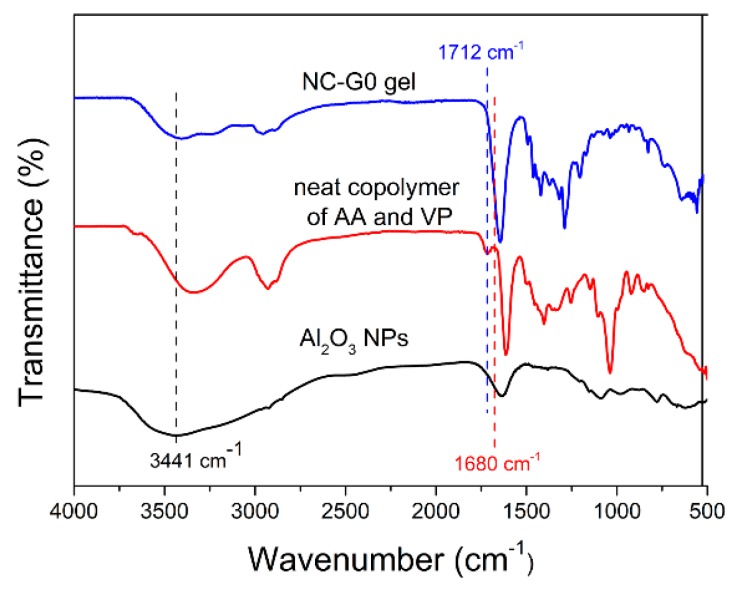
Fourier transform infrared (FTIR) spectra of alumina nanoparticles (Al_2_O_3_ NPs), neat copolymer and NC-G0 gel.

Figure 2 displays the ATR-FTIR spectra of Al_2_O_3_ NPs, neat copolymer of AA and VP, as well as dried NC-G0 gel. The disappearance of the band at 1680 cm^-1^ (characteristic band of vinyl groups) in the spectra of neat copolymer and NC-G0 gel indicates that almost all the monomers participated in the polymerization during the UV irradiation. In addition, this result also verifies that the incorporation of Al_2_O_3_ NPs would not hinder the copolymerization of AA and VP, which is an important prerequisite for the formation of hydrogels. Furthermore, compared to the spectra of neat copolymer and that of Al_2_O_3_ NPs, the disappearance of the peak of 1712 cm^−1^ (characteristic band of –COOH) as well as the weakening of peak centered at 3441 cm^−1^ (characteristic band of –OH) in the spectrum of NC-G0 gel imply that a strong interaction exists between the carboxyl groups and Al_2_O_3_ NPs. According to the literature [22,26,27,28], these interactions probably correspond to the topotactic chelation reactions between the dissociated carboxyl groups (–COO^–^) on the polymer chains and the aluminum atoms on the surface of Al_2_O_3_ NPs (Scheme 1e), which contributed to the cross-linking structures of NC-G gels. This result verifies that the Al_2_O_3_ NPs acted as cross-linking agents, since extra organic cross-linkers were involved in the preparation procedure [22].

### 3.2. Anti-Dehydration and Swelling Resistant Properties of NC-G Gels

**Figure 3:**
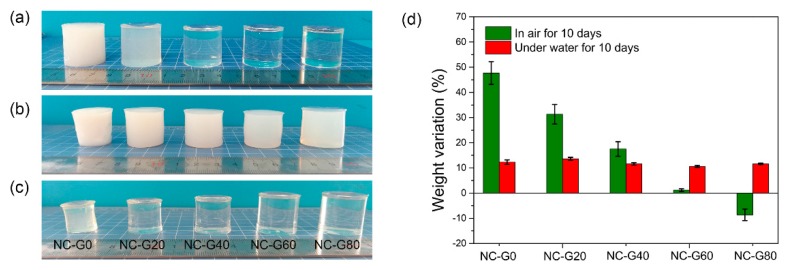
The digital pictures of NC-G gels. (**a**) As-prepared, (**b**) immersed in water for 10 days, (**c**) placed in air for 10 days, (**d**) weight variation of NC-G gels.

The evaporation of water in air and absorbing of water in water environments are two major and long-lasting problems that limit the practical application of hydrogels because of their great influence on the mechanical properties of hydrogels. Our previous report demonstrated that hydrogels with low swelling ratios can be obtained by using AA and *N*,*N*-dimethylacrylamide (DMAA) as the co-monomer, and Al_2_O_3_ NPs as the inorganic cross-linkers due to the strong chelation reactions between polymer chains and nanoparticles [22]. Recently, Lu et al. reported that the incorporation of glycerol in hydrogels could prevent the loss of water and maintain the properties of the hydrogel in a wide temperature range from −20 to 60 °C because of the strong hydrogen bonding interactions between water and glycerol [29]. Based on these reports, we thought that the newly prepared NC-G gels could possess both swelling resistant and anti-dehydration properties. To verify this hypothesis, hydrogels with different glycerol content were placed in air and under water for 10 days to observe their changes in volume and weight. Figure 3a shows the digital pictures of as-prepared NC-G gels with different glycerol contents. The NC-G0 gel, which contained no glycerol, is opaque, which is different from the hydrogels prepared form AA and DMAA [22]. It has been widely acknowledged that the carbonyl groups on the VP segment could form hydrogen bonds with the carboxyl groups introduced by AA [30,31]. These hydrogen bonds would probably cause the formation of small polymer aggregates within hydrogels, which leads to the phase separation, thus resulting in the opaque appearance of NC-G0 gels [32]. This can be verified by the transparent appearance of Al_2_O_3_ cross-linked hydrogels using AA and DMAA as the monomer with the same monomer ratio, in which the hydrogen bonding between the polymer chains is much lower than that in the NC-G0 gel, probably due to the steric hindrance of the two methyl groups. However, with the incorporation of glycerol, a well-known plasticizer, into the hydrogel system, the hydrogels gradually transformed from opaque to totally transparent. This is because the glycerol was able to destroy the hydrogen bonds between polymer chains, preventing the formation of polymer aggregates. Therefore, the transparence of the hydrogels increased with increasing glycerol content.

Distinct from most of the hydrogels that would absorb water to swell under aqueous environments, the NC-G gels not only did not swell, but even slightly shrunk after immersion in water for 10 days, as revealed in Figure 3b. The results from the quantitative measurements show that all of the hydrogels shrank about 10% (*w*/*w*) compared to their original weight (Figure 3d). This phenomenon can be explained based on the cross-linking mechanism of Al_2_O_3_ NPs cross-linked hydrogels. As we discussed previously, the cross-linkage within NC-G gels contributed to the interactions between –COO^–^ groups on polymer chains and Al_2_O_3_ NPs. In our experiment, the Al_2_O_3_ NPs were dispersed in acidic media to result in a transparent Al_2_O_3_ colloid solution with a pH value around four. After polymerization, the liquid phase within the hydrogels was also acidic, with a pH value lower than the pKa (4.75) of –COOH. Under this condition, only part of the –COOH on the polymer chains deprotonated to –COO^–^, which could interact with Al_2_O_3_ NPs to form cross-linkage. However, when NC-G gels were immersed in neutral deionized water, more –COO^–^ groups can be generated to react with Al_2_O_3_ NPs during the solvent exchange process, which could increase the cross-linking density of hydrogels, leading to the shrinkage of the hydrogels. Furthermore, from Figure 3b, all the hydrogels became opaque again after immersion in water for 10 days. This is because the replacement of glycerol with water eliminated the plasticizing effect of glycerol; therefore, the phase separation occurred again and the hydrogel turned opaque.

**Figure 4:**
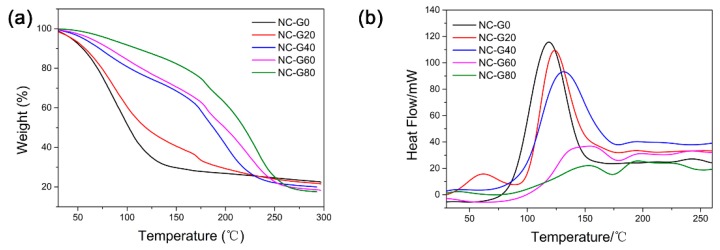
(**a**) Thermal gravimetric analysis (TGA) and (**b**) differential scanning calorimetry (DSC) curves of NC-G gels.

Figure 3c displays the pictures of NC-G gels placed in air after 10 days. It can be seen that the NC-G0 gel shrank significantly due to the evaporation of water. However, when glycerol was incorporated, the volume change became increasingly less obvious. According to the quantitative data shown in Figure 3d, the weight variation gradually decreased from about 47.5% to 1.2% as the glycerol content increased from 0% to 60%. As for the NC-G80 gel, the sample even absorbed water from the environment. This result confirms that the incorporation of glycerol could indeed inhibit the water evaporation of water within hydrogels due to the strong hydrogen bonding interactions between glycerol and water, as well as the lower volatility and a higher boiling point of glycerol [29]. To prove this conclusion, TGA and DSC were used to characterize the NC-G gels and the results are shown in Figure 4. It can be seen from Figure 4a that the NC-G0 gel lost more than 40% of its original weight at 100 °C due to the evaporation of water. However, when glycerol was introduced, higher temperatures were needed to remove the liquid from the NC-G gels. From the DSC curves (Figure 4b), the endothermic peaks shifted from 118.7 °C to 156.1 °C as the glycerol content increased from 0% to 80%. All these results confirm that the introduction of glycerol in the hydrogel systems could delay the evaporation of the liquid from the hydrogels, thus imparting the hydrogels anti-dehydration properties.

### 3.3. Mechanical Properties of NC-G Gels

**Figure 5:**
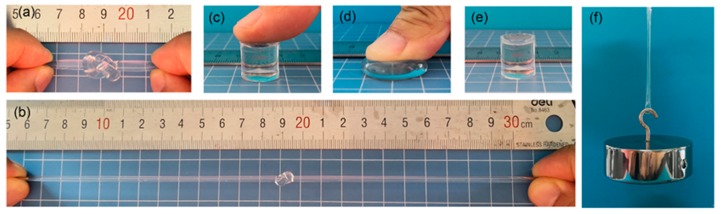
Illustration of the outstanding mechanical performance of NC-G gels. (**a**) Knotted, (**b**) stretch after knotting, (**c**) before compression, (**d**) under compression, (**e**) after compression, and (**f**) lift up a 1.0 kg weight.

The mechanical properties of hydrogels play a key role in their practical applications. The currently reported NC-G gels with dehydration and swelling-resistant properties also had outstanding mechanical performance. As displayed in Figure 5, the as-prepared NC-G60 gel could withstand large-scale deformations, e.g., stretch after knotting and compression without breaking. Furthermore, a hydrogel strip with a diameter of 5.5 mm could lift up to a 1.0 kg weight, showing excellent mechanical strength. In contrast, the OR-G60 gel cross-linked by organic cross-linker with the same monomer and glycerol content as NC-G60 gel were fragile and brittle, which was unable to withstand the deformations mentioned above.

**Figure 6:**
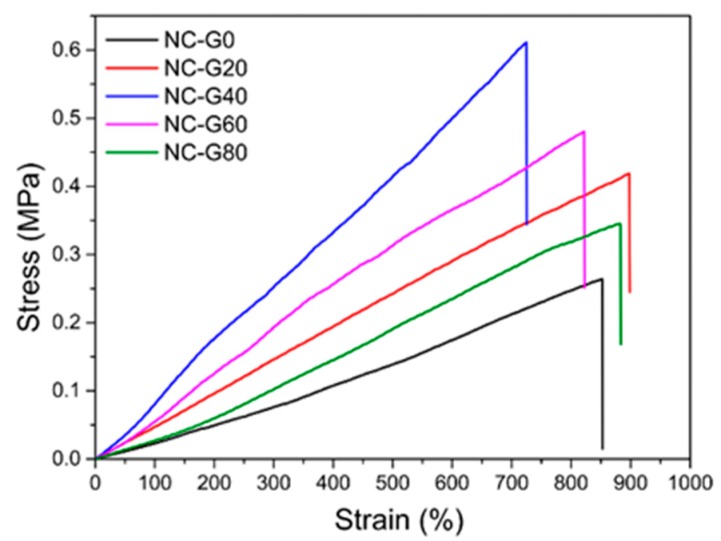
Stress-strain curves of NC-G gels with different glycerol content.

To quantitatively measure the mechanical properties of NC-G gels, uniaxial tensile tests were conducted and the stress and strain curves of hydrogels are shown in Figure 6. The results indicate that all the prepared NC-G gels could be stretched more than seven times their original length with a tensile strength between 0.26 to 0.61 MPa, which is comparable to nanocomposite hydrogels cross-linked by other inorganic nanomaterials [14,33,34]. Here, it is worth noting that the hydrogels precursors only contained 2% Al_2_O_3_ NPs, so the mechanical performance of NC-G gels could be further enhanced by increasing the Al_2_O_3_ concentration in the precursor solution. Besides, the glycerol content could affect the mechanical properties of hydrogels. As shown in the figure, the tensile strength gradually increased as the glycerol content increased from 0% to 40%, after which the strength started to decrease. The reason for this phenomenon may be explained as follows. (1) As mentioned above, the incorporation of glycerol in the hydrogel systems could destroy the hydrogen bonds between polymer chains, which increases the flexibility of the polymer chains. (2) It has been reported that the glycerol-water mixtures exhibit stronger interactions with the polymers than pure water, which could introduce more noncovalent interactions in the polymer networks acting as sacrificial bonds to improve the toughness of NC-G gels [29]. (3) The glycerol could form hydrogen bonds with the carboxyl groups on the polymer chain, which would lead to the reduced cross-linking density of hydrogels, since the cross-linkage of NC-G gels contributed to the chelation reactions between Al_2_O_3_ NPs and carboxyl groups. These three factors could influence the mechanical properties of NC-G gels. When the glycerol content was under 40%, the strong interactions of the glycerol-water mixtures and polymer networks were dominant; therefore, the mechanical strength increased with increasing glycerol content. However, when the glycerol content was higher than 40%, the hydrogen bonds between glycerol and carboxyl groups caused the decrease of the cross-linking density of NC-G gels. As a result, the mechanical properties decreased accordingly.

**Figure 7:**
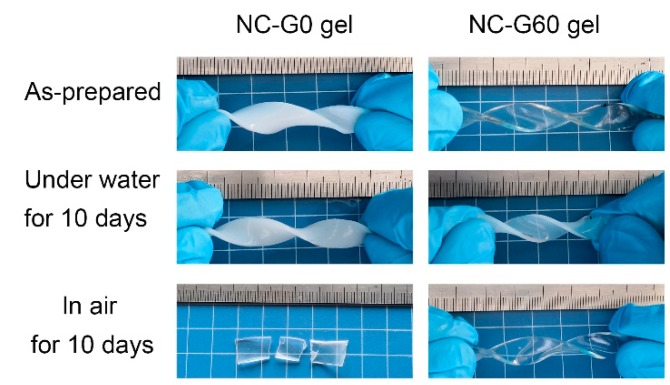
Illustration of NC-G gels at different conditions.

**Figure 8:**
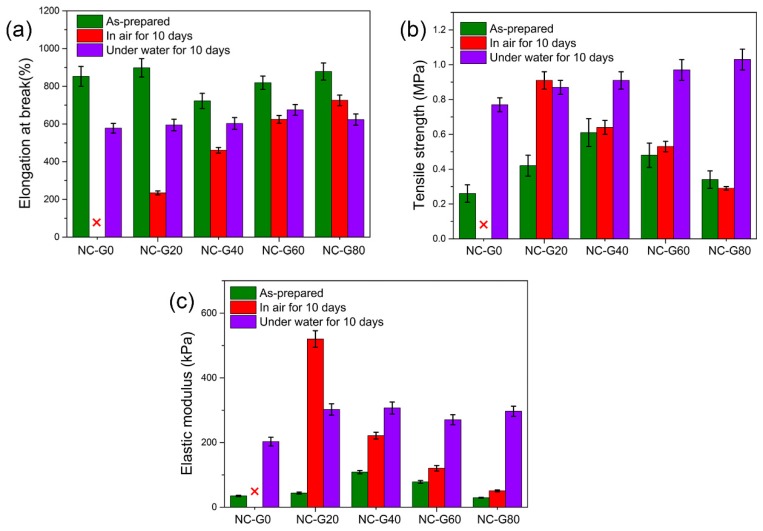
Comparison of mechanical properties of NC-G gels placed in air and under water for 10 days. (**a**) Elongation at break; (**b**) Tensile strength; (**c**) Elastic modulus.

Due to the anti-dehydration and swell-resistant properties as well as the excellent mechanical properties of their as-prepared states, we thought that the NC-G gels could also possess considerable mechanical performance both in air and water environments. Therefore, mechanical tests were conducted on hydrogels after being placed either in air for 10 days or immersed in water for 10 days. As shown in Figure 7, the NC-G0 gels exhibited excellent mechanical toughness after immersion in water for 10 days, and could withstand twisting without breaking. However, after being placed in air for 10 days, the hydrogel became stiff and brittle because of the loss of water within the hydrogels. Thus, the hydrogel was easily fractured upon deformation. In contrast, the NC-G60 gel maintained its outstanding mechanical performance both in air and water, which simultaneously overcame the two long-lasting problems inhibiting the practical applications of hydrogel materials. Based on these findings, we also measured the mechanical properties of hydrogels under different conditions, and the results are summarized in Figure 8. All of the NC-G gels demonstrated similar mechanical properties after immersion in water for 10 days. This is because the glycerol within hydrogels was replaced with water, which causes the different NC-G gels to have similar compositions after immersion in water. Furthermore, as discussed above, the volumes of the NC-G gels shrank 10% after immersion in water due to the increased cross-linking density. Therefore, the tensile strength and elastic moduli of the hydrogels significantly increased, while the elongations at break slightly decreased. Notably, the tensile strength of the hydrogels immersed in water were as high as 1 MPa, making these gels prospective for application in load-bearing systems. As for the hydrogels placed in air for 10 days, their mechanical properties revealed a significant relationship with their glycerol content. For the NC-G0 gel, most of the water evaporated during the 10 days. Thus, no mechanical data could be obtained due to its stiff and fragile nature. When 20% of the water was replaced by glycerol, the NC-G20 gel was still soft after being placed in air for 10 days. It could be stretched more than 200% (234 ± 10.2%) its original length before fracture because of the reduced water loss from the hydrogels, showing tensile strength and elastic modulus around 0.91 ± 0.05 MPa and 520.2 ± 25.6 kPa, respectively. Further increasing the glycerol content led to the increasing in the softness and extensibility of the resultant hydrogels, whereas the elastic modulus gradually decreased. In addition, the tensile strength of NC-G40 gel, NC-G60 gel, and NC-G80 gel after being placed in air for 10 days were measured to be 0.64 ± 0.04 MPa, 0.53 ± 0.03 MPa, and 0.29 ± 0.01 MPa, respectively. These values are almost the same as those recorded in their as-prepared states and comparable to other high-strength hydrogels reported in the literature [33,34,35]. In general, the data displayed in Figure 8 strongly verified that the NC-G gels could be utilized in air and water environments because of their excellent mechanical performance under these conditions.

## 4. Conclusions

In summary, we prepared a novel type of high-strength nanocomposite hydrogel with anti-dehydration and swelling-resistant properties. The hydrogels were prepared by *in situ* free radical copolymerization of AA and VP in mixtures of water and glycerol using Al_2_O_3_ NPs as the inorganic cross-linking agents. The topotactic chelation reactions between Al_2_O_3_ NPs and the polymer matrix not only contributed to the outstanding mechanical properties of hydrogels, but also endowed them with swelling resistance. Simultaneously, the strong hydrogen bonding interactions between water and glycerol were responsible for the anti-dehydration capacity of the resultant hydrogels. Based on these unique properties, the prepared hydrogels maintained high mechanical performance both in air and water environments, which would largely enhance the practical applications of hydrogels.

## Figures and Tables

**Table 1:** Compositions of nanocomposite hydrogels (NC-G gels) and OR-G gel.

Sample	Glycerol (g)	Water (g)	AA (g)	VP (g)	Al_2_O_3_ (g)	MBA (mg)
NC-G0	0	10	0.63	2.33	0.2	0
NC-G20	2	8	0.63	2.33	0.2	0
NC-G40	4	6	0.63	2.33	0.2	0
NC-G60	6	4	0.63	2.33	0.2	0
NC-G80	8	2	0.63	2.33	0.2	0
OR-G60	6	4	0.63	2.33	0	30

Note: 20 mg Irgacure 2959 was used in all the samples as the photo-initiator; AA: acrylic acid; VP: *N*-vinyl-2-pyrrolidone; MBA: *N*,*N*′-methylenebis-(acrylamide).
